# Patterns of sex-specific and age-specific risk indicators of suicide: a population-nested case-control study

**DOI:** 10.1136/bmjment-2025-301959

**Published:** 2025-10-28

**Authors:** Fred Johansson, Lisa Gunnarsson, Leoni Grossmann, David Mataix-Cols, Lorena Fernández de la Cruz, Seena Fazel, Renee M Gardner, Christina Dalman, John Wallert, Christian Rück

**Affiliations:** 1 Centre for Psychiatry Research, Department of Clinical Neuroscience, Karolinska Institutet, & Stockholm Health Care Services, Region Stockholm, Stockholm, Sweden; 2 Deparment of Psychiatry, University of Oxford, Oxford, England, UK; 3 Department of Clinical Sciences, Lund University, Lund, Sweden; 4 Oxford Health NHS Foundation Trust, Oxford, England, UK; 5 Department of Global Public Health, Karolinska Institutet, Stockholm, Sweden; 6 Center for Epidemiology and Community Medicine, Stockholm, Sweden

**Keywords:** Psychiatry

## Abstract

**Background:**

Suicide is more common among males and in older age, but the understanding of sex-specific and age-specific risk indicators is limited.

**Objective:**

To describe the sex-specific and age-specific prevalence of 25 suicide risk indicators in the year preceding suicide and estimate their associations with suicide.

**Methods:**

Register-based population-nested case-control study in Sweden, 2009–2021, comprising 19 741 suicide cases and 197 296 general population controls matched by sex, age and county of residence. Death by suicide was collected from the cause of death register. 25 suicide risk indicators covering psychiatric history, somatic disorders, bereavement and sociodemographic factors in the previous year were collected from nationwide registers. Sex-specific and age-specific ORs of suicide for the presence/absence of each risk indicator in the prior year were estimated and complemented by risk differences.

**Findings:**

Suicide cases were 70% male, 9% were aged 15–24 years, 29% 25–44 years, 36% 45–64 years and 26% 65+ years. In the year preceding suicide, the prevalence of most risk indicators was the lowest among males and people aged 65+ years. Most risk indicators also showed weaker 1-year associations with suicide in these groups. The median OR (IQR) of suicide across the 25 risk indicators was 14.6 (5.2, 29.1) in females versus 10.3 (4.3, 21.3) in males, and 17.4 (6.5, 28.9) in 24–44 year-olds versus 8.0 (3.6, 23.7) in people aged 65+years. Risk differences of suicide were larger in males across nearly all risk indicators.

**Conclusions:**

There was considerable heterogeneity across sex and age groups, both in the prevalence of risk indicators preceding suicide and in their associations with suicide. Risk indicators were generally less common and displayed weaker associations with suicide on the relative risk scale among males and older people.

**Clinical implications:**

Suicides in males and older people may be harder to predict, as indicators are rarer. When males present with risk indicators, they generally have a higher absolute risk of suicide, making them important targets for prevention even when risk indicators do not cause suicide. Our findings underscore the importance of considering sex-specific and age-specific risk indicators for individualised suicide prediction and prevention.

**WHAT IS ALREADY KNOWN ON THIS TOPIC**

- Suicide rates are twice as high among males as among females and increase with age, but comprehensive reports on sex-specific and age-specific suicide risk indicators are largely lacking.

**WHAT THIS STUDY ADDS**

- Our results show that male and older suicide cases had a lower prevalence of most risk indicators in the year preceding suicide. Most risk indicators also showed weaker associations with suicide among males and older people than among females and younger people on the relative risk scale.

**HOW THIS STUDY MIGHT AFFECT RESEARCH, PRACTICE OR POLICY**

- Suicide among males and older people may be harder to predict given the relative lack of strong risk indicators preceding suicide in these groups, calling for sex-tailored and age-tailored suicide prediction and prevention.

## Background

 Suicide is a major public health concern accounting for over 700 000 deaths worldwide each year.^1^ The global suicide incidence is about twice as high among males as among females.^2^ In contrast, key suicide risk indicators, such as self-harm and mental disorders,^3 4^ are not only more prevalent among females,^5^,^7^ but also show stronger associations with suicide on the relative risk scale.^8^,^11^ Similar paradoxical findings pertain to age. Suicide rates are the highest in middle-aged and older age groups,^2^ yet key risk indicators like self-harm and psychiatric hospitalisations are more common among younger people.^12^

Most studies on sex-specific and age-specific suicide risk indicators focus on only one or a few exposures or are restricted to certain sex or age groups. Such a narrow focus may fail to uncover general patterns of risk indicators over the life course. Further, suicide risk and most of its determinants vary over time, with the highest risk in close temporal proximity to exposure.^4 9 13^ The use of different exposure windows and follow-up times makes it difficult to compare risk indicators between studies. Long follow-up times and static measures of risk factors have been identified as major limitations of suicide risk factor research,^14^ precluding conclusions on dynamic aspects of suicide risk and risk indicators over the life course. Identifying sex-specific and age-specific patterns across a wide range of suicide risk indicators could help delineate sensitive periods for specific risk indicators and provide insights into sex- and age-based heterogeneity in processes leading up to suicide. Thus, there is a clear need for research comparing different suicide risk indicators across sex and age groups within a short timeframe.

## Objective

In the current study, we aim to address these issues by providing a comprehensive account of short-term suicide risk indicators across sex and age groups. We define risk indicators as variables denoting an increased risk of suicide, not as necessarily causal risk factors. We use data from Swedish registers to describe (1) the sex-specific and age-specific prevalence of 25 clinical and sociodemographic risk indicators in the year preceding suicide and (2) their one year association with suicide in the general population.

## Methods

### Design and population

The source population consisted of all people aged 15 or older residing in Sweden at any time between 1 January 2009 and 31 December 2021. We used a population-nested case-control design that provided estimates approximating hazard ratios, or risk ratios, for rare outcomes such as suicide.^15^ Suicide cases were defined as all persons dying by suicide during the study period, as recorded in the cause of death register (CoDR). Controls were sampled from all individuals alive in the population at the index date, that is, the death date of their case counterparts (incidence-density sampling) and matched to cases on age, sex and county of residence at death of the case in a 1:10 ratio using the Total Population Register.

Data from Swedish administrative registers were linked using the de-identified Swedish personal identification number. Additional registers used were the National Patient Register (NPR) including healthcare data registered by physicians in inpatient and specialised outpatient care; the Longitudinal Integrated Database for Health Insurance and Labour Market Studies (LISA); the Prescribed Drug Register (PDR); the Cancer Register (CR) and the Multi-Generation Register (MGR). Ethical approval was granted by the Swedish Ethical Review Authority (2010-1185-31-5, 2022-02662-02), without requiring informed consent due to data pseudonymisation.

### Outcome

Death by suicide was defined as death by either intentional self-harm (ICD-10: X60–X84) or self-harm with undetermined intent (ICD-10: Y10-34) as the underlying or contributing cause of death in the CoDR.

### Exposure: risk indicators

Risk indicators were selected based on prior research and availability in the Swedish registers, aiming to cover main domains of suicide risk indicators.^3 4 16^ To capture short-term risk indicators, an exposure window of 1 year prior to the index date was used. This exposure window was chosen to capture short-term fluctuations in suicide risk, while still providing enough statistical power to detect associations even for rare risk indicators (for details, see the power calculations in the pre-registered analysis plan (https://osf.io/2dv3f). We included 25 risk indicators across six domains*: **Self-harm and psychiatric contacts**: psychiatric inpatient care, psychiatric outpatient care* and *intentional self-harm* (International Classification of Diseases, 10th Revision [ICD-10]: X60-84) as registered in the NPR; ***Mental disorder diagnoses**: anxiety disorders* (ICD-10: F40-41), *depressive disorders* (ICD-10: F32-39 excl. F32.3), *psychotic disorders* (ICD-10 codes: F20-29), *personality disorders* (ICD-10: F60-61)*, stress-related disorders* (F43) and *substance-use disorders* (ICD-10: F10-19), as registered in the NPR; ***Psychotropic medications**:* any dispensation of *antidepressants* (ATC: N06A), *antipsychotics* (ATC: N05A), *psychostimulants* (ATC: N06B) and *sedatives* (ATC: N05B-C) as registered in the PDR; ***Somatic diseases*** include *aggressive cancers* (defined by the 5-year survival rate in line with prior research^17^; ICD-10: C15, C22-C25, C33-C34, C56, C76, C80), *amyotrophic lateral sclerosis* (ICD-10: G12.2) and *dementia and delirium not induced by alcohol or drugs* (ICD-10: F00-F09, G30-31), as registered in from the CR and the NPR; ***Bereavement**: death of a child (<18 years), death of a child (≥18 years*) and *death of a first degree relative*, derived by combining data from the MGR and the CoDR; ***Sociodemographic factors**: divorce*, *unemployment* (>180 days of unemployment in the prior calendar year)*, long-term sickness absence* (>90 net days of sickness absence in the prior year), *disability pension*, *relative poverty* (≤60% of the family-adjusted median income) and receipt of *social benefits*, as registered in LISA. Indicators related to working life (eg, unemployment) were not used for people 65+ years. For the sociodemographic factors, only persons >16 years of age were included in the analyses, since prior year information was not available for 15-year-olds. Risk indicator details are provided in online supplemental eTable 1.

### Statistical analysis

All analyses were stratified by sex (female/male) and age group (15–24 years, 25–44 years, 45–64 years and 65+ years). Risk indicator prevalence is presented as counts with percentages, with the case prevalence equalling the sensitivity of the risk indicator. The relative risk of suicide in the year following each risk indicator was estimated using conditional logistic regression and presented as OR with 95% CI for each sex and age strata. We used an exposure window of 1 year, which approximates a cohort study with time-varying exposures^18^ in which people are considered exposed up to 1 year after the last registration of the risk indicator. Ties within the risk sets were handled using the ‘exact method’.^19^ Separate models were fitted for each risk indicator in each sex/age strata and were not adjusted for any covariates other than the matching variables (sex, age and county of residence). Since each risk indicator was modelled separately, estimates are not independent in relation to the other risk indicators. Phi correlations between the risk indicators are presented in online supplemental eFigure 1. Risk differences, defined as the difference in suicide rate per 100 000 persons years between exposed and unexposed, were approximated by transforming ORs using formulas presented by Greenland.^20^ For these transformations, suicide base rates for each sex and age strata were collected from publicly available CoDR data for the full Swedish population. Since controls were matched to cases on sex, age and county of residence, we could not estimate ORs or risk differences for these variables.

We had no missing data on the risk indicator variables, except that the sociodemographic variables were missing for 64 (<0.1%) people. All analyses were performed on complete case data. Analyses were performed in R V.4.3.2 using the packages *Epi* and s*urvival*. A preregistration of the analytic plan is available at https://osf.io/2dv3f.

#### Sensitivity analysis

People may self-harm and die from injury days later. For them, diagnoses and self-harm recorded prior to their death may relate to the same self-harm event that is later coded as suicide. To avoid reverse causation by events directly linked to the suicide episode, we merged all consecutive inpatient stays one day or less apart (to account for people moving between wards and hospitals) and retained risk indicators only if people had been discharged for more than a week prior to their index date.

## Findings

We included 19 741 individuals who died by suicide from 2009 through 2021 and 197 296 general population controls. Most that died were male (70%), and most frequently between 45–64 years (36%). Suicide cases had lower income and lower education than controls, and markedly more psychiatric outpatient and inpatient care (table 1).

**Table 1:** Characteristics of suicide cases and matched general population controls

	Cases (n=19 741)	Controls* (n=197 296)
Age group, n (%)		
15–24 years	1739 (8.8)	17 291 (8.8)
25–44 years	5659 (28.7)	56 586 (28.7)
45–64 years	7166 (36.3)	71 650 (36.3)
65+ years	5177 (26.2)	51 769 (26.2)
Male (%)	13 718 (69.5)	137 112 (69.5)
Income category, n (%)		
Low (quintile 1)	8589 (43.5)	38 012 (19.3)
Middle (quintile 2–4)	9315 (47.2)	119 912 (60.8)
High (quintile 5)	1778 (9.0)	39 367 (20.0)
Missing	59 (0.3)	5 (0.0)
Education, n (%)		
Elementary (≤9 years or less)	5874 (29.8)	42 076 (21.3)
Secondary (10–12 years)	4152 (21.0)	63 030 (31.9)
Higher (>12 years)	9401 (47.6)	88 616 (44.9)
Missing	314 (1.6)	3574 (1.8)
Prior psychiatric outpatient care, n (%)	11 694 (59.2)	19 669 (10.0)
Prior psychiatric inpatient care, n (%)	10 289 (52.1)	11 940 (6.1)

* Controls are matched on sex, age (in years) and county of residence.

### Prevalence of risk indicators preceding suicide

Of those who died by suicide, 57% were dispensed sedatives and 49% antidepressants in the preceding year, compared with 12% and 10% of controls, respectively; 42% had psychiatric outpatient visits, compared with 3% of controls; 25% had received psychiatric inpatient care versus 0.6% among controls and 10% had records of self-harm in the year preceding suicide compared with 0.1% among controls (online supplemental eTable 2).

**Figure 1 F1:**
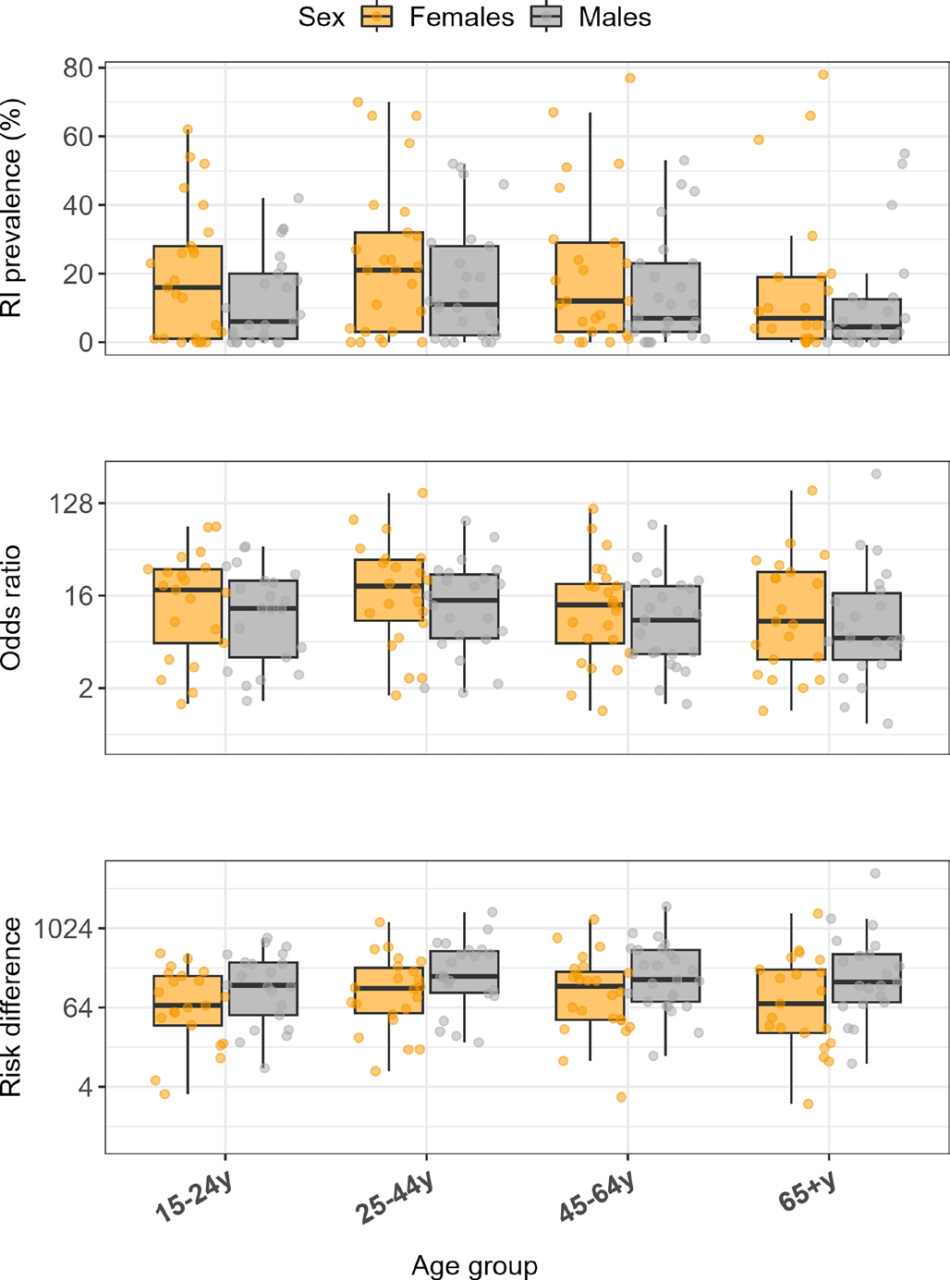
Distribution of the risk indicator prevalence and associations with suicide for the 25 risk indicators across sex and age groups. Upper panel: boxplots of risk indicator prevalence (%) for each of the 25 risk indicators in the year preceding suicide by sex and age group strata of 19 741 suicide cases (estimates presented in table 2). Middle panel: ORs between of suicide between exposed and unexposed by sex and age group strata in 19 741 suicide cases and 197 296 controls (estimates presented in table 3). Lower panel: risk differences, measured as the difference of suicide rates per 100 000 person-years between exposed and unexposed (estimates presented in online supplemental eTable 3). RI: risk indicator.

Nearly all risk indicators were more prevalent among females that died by suicide compared with males (table 2, figure 1). For those that died by suicide, the median point prevalence (IQR) across the 25 risk indicators was 13% (1%, 29%) in females and 7% (2%, 20%) in males. In the year preceding suicide, females had a higher occurrence of self-harm, psychiatric inpatient and outpatient care, all mental disorders except substance use and all sociodemographic factors except unemployment. Most risk indicators also occurred more frequently in the age group 25–44 years and decreased with age (table 2, figure 1). The median point prevalence (IQR) was 12% (1%, 26%) in 15–24 year-olds, 16% (3%, 30%) in 25–44 year-olds, 11% (3%, 24%) in 45–64 year-olds and 5% (1%,16%) in those 65 years or above that died by suicide. Some notable exceptions to this age trend were antidepressants, sedatives and relative poverty, for which the prevalence remained largely stable or increased with age.

**Table 2:** Prevalence of specific risk indicators in the year preceding suicide among 19 741 suicide cases (corresponding table for controls in online supplemental eTable 4)

Risk indicator prevalence in suicide cases, N (%)								
	15–24 years		25–44 years		45–64 years		65+ years	
	Female (n=520)	Male (n=1219)	Female (n=1627)	Male (n=4032)	Female (n=2279)	Male (n=4887)	Female (n=1597)	Male (n=3580)
Self-harm and psychiatric contacts								
Self-harm	137 (26.3)	119 (9.8)	343 (21.1)	433 (10.7)	283 (12.4)	341 (7.0)	153 (9.6)	198 (5.5)
Psychiatric inpatient care	207 (39.8)	300 (24.6)	648 (39.8)	1211 (30.0)	650 (28.5)	1120 (22.9)	296 (18.5)	459 (12.8)
Psychiatric outpatient care	322 (61.9)	508 (41.7)	1075 (66.1)	2054 (50.9)	1164 (51.1)	1877 (38.4)	499 (31.2)	721 (20.1)
Mental disorders								
Anxiety disorder	167 (32.1)	210 (17.2)	517 (31.8)	750 (18.6)	481 (21.1)	639 (13.1)	241 (15.1)	252 (7.0)
Depressive disorder	145 (27.9)	190 (15.6)	436 (26.8)	748 (18.6)	549 (24.1)	795 (16.3)	301 (18.8)	451 (12.6)
Personality disorder	91 (17.5)	31 (2.5)	344 (21.1)	239 (5.9)	167 (7.3)	131 (2.7)	19 (1.2)	14 (0.4)
Psychotic disorder	27 (5.2)	68 (5.6)	144 (8.9)	399 (9.9)	171 (7.5)	289 (5.9)	82 (5.1)	65 (1.8)
Stress-related disorder	84 (16.2)	67 (5.5)	273 (16.8)	315 (7.8)	240 (10.5)	313 (6.4)	68 (4.3)	105 (2.9)
Substance use disorder	139 (26.7)	270 (22.1)	501 (30.8)	1153 (28.6)	526 (23.1)	1109 (22.7)	162 (10.1)	330 (9.2)
Psychotropic medication								
Antidepressant	280 (53.8)	400 (32.8)	1077 (66.2)	1847 (45.8)	1524 (66.9)	2136 (43.7)	938 (58.7)	1419 (39.6)
Antipsychotic	137 (26.3)	216 (17.7)	624 (38.4)	1128 (28.0)	682 (29.9)	938 (19.2)	327 (20.5)	401 (11.2)
Psycho stimulants	70 (13.5)	94 (7.7)	181 (11.1)	391 (9.7)	95 (4.2)	144 (2.9)	<10	19 (0.5)
Sedatives	271 (52.1)	395 (32.4)	1134 (69.7)	1989 (49.3)	1744 (76.5)	2583 (52.9)	1241 (77.7)	1976 (55.2)
Somatic conditions								
Aggressive cancers	<10	<10	<10	<10	<10	<10	16 (1.0)	39 (1.1)
Amyotrophic lateral sclerosis	<10	<10	<10	<10	<10	<10	<10	<10
Dementia or delirium	<10	<10	20 (1.2)	23 (0.6)	34 (1.5)	67 (1.4)	78 (4.9)	130 (3.6)
Bereavement								
Death of a first degree relative	<10	16 (1.3)	52 (3.2)	96 (2.4)	133 (5.8)	317 (6.5)	60 (3.8)	99 (2.8)
Death of a child (<18 years)	<10	<10	<10	<10	<10	<10	<10	<10
Death of a child (*≥* 18 years)	<10	<10	<10	<10	14 (0.6)	<10	12 (0.8)	13 (0.4)
Sociodemographic factors*								
Disability pension	70 (13.7)	63 (5.2)	384 (23.7)	579 (14.4)	1027 (45.1)	1329 (27.2)	140 (8.8)	181 (5.1)
Divorce	<10	<10	41 (2.5)	64 (1.6)	46 (2.0)	117 (2.4)	<10	20 (0.6)
Long-term sickness absence	17 (3.3)	28 (2.3)	360 (22.2)	489 (12.2)	410 (18.0)	553 (11.3)	NA^†^	NA^†^
Relative poverty	229 (44.8)	394 (32.7)	943 (58.2)	2072 (51.5)	1188 (52.2)	2251 (46.1)	1048 (65.7)	1863 (52.1)
Social benefits	117 (22.9)	247 (20.5)	385 (23.8)	915 (22.7)	283 (12.4)	549 (11.3)	NA^†^	NA^†^
Unemployment	<10	22 (1.8)	71 (4.4)	257 (6.4)	73 (3.2)	240 (4.9)	NA^†^	NA^†^

* The LISA register contains information on people from 15 years of age, decedents of age 15 years were excluded since prior year information was not available.

† Risk indicators related to working life excluded for people 65+ years.

LISA, Longitudinal Integrated Database for Health Insurance and Labour Market Studies.

### Associations between risk indicators and suicide

The 25 risk indicators were associated with suicide across nearly all sex and age strata, but the strength of the associations varied between sex and age groups. Overall, self-harm and psychiatric inpatient care were the strongest risk indicators of suicide, followed by specific mental disorders and outpatient psychiatric care, particularly personality disorder and substance use disorder diagnoses (table 3, unstratified estimates in online supplemental eTable 2). Although these top risk indicators were largely invariant across sex and age groups, the magnitudes differed.

**Table 3 T3:** ORs of suicide between exposed and unexposed conditional on sex and age groups in the general population

OR* (95% CI)								
	15–24 years		25–44 years		45–64 years		65+ years	
	Female	Male	Female	Male	Female	Male	Female	Male
Self-harm and psychiatric contacts								
Self-harm	74.4 (45.5 to 122)	47.4 (30.8 to 73.0)	161 (104 to 250)	85.8 (64.0 to 115)	113 (74.9 to 170)	78.9 (57.5 to 108)	170 (86.8 to 333)	247 (123 to 501)
Psychiatric inpatient care	75.6 (50.2 to 114)	48.3 (36.6 to 63.8)	88.4 (69.1 to 113)	59.7 (51.3 to 69.5)	72.2 (57.8 to 90.3)	37.9 (33.3 to 43.2)	52.0 (39.0 to 69.3)	44.3 (35.7 to 54.9)
Psychiatric outpatient care	22.6 (18.0 to 28.5)	14.1 (12.1 to 16.3)	36.7 (31.7 to 42.5)	26.6 (24.3 to 29.2)	28.8 (25.5 to 32.6)	18.8 (17.3 to 20.4)	23.7 (20.0 to 28.0)	18.0 (15.8 to 20.4)
Mental disorders								
Anxiety disorder	18.2 (13.8 to 23.9)	21.4 (16.7 to 27.4)	26.7 (22.4 to 31.7)	20.8 (18.2 to 23.6)	19.9 (16.9 to 23.3)	20.2 (17.7 to 23.2)	23.7 (18.7 to 30.0)	20.8 (16.7 to 25.9)
Depressive disorder	20.0 (14.8 to 27.0)	15.7 (12.4 to 19.8)	18.7 (15.9 to 22.1)	22.5 (19.7 to 25.7)	23.6 (20.1 to 27.6)	19.8 (17.5 to 22.4)	27.2 (21.7 to 34.1)	26.0 (21.7 to 31.0)
Personality disorder	42.8 (26.6 to 68.8)	25.8 (13.3 to 50.3)	46.2 (35.9 to 59.6)	28.5 (22.2 to 36.6)	29.4 (21.7 to 39.8)	20.9 (15.5 to 28.3)	31.7 (12.6 to 79.3)	14.0 (6.2 to 31.5)
Psychotic disorder	27.0 (13.1 to 55.8)	33.8 (20.5 to 55.6)	30.3 (21.8 to 42.2)	23.5 (19.6 to 28.1)	13.4 (10.6 to 17.0)	9.2 (7.8 to 10.8)	11.7 (8.5 to 16.1)	8.0 (5.8 to 11.2)
Stress-related disorder	25.1 (16.7 to 37.7)	18.8 (12.5 to 28.3)	21.0 (17 to 26.1)	19.7 (16.2 to 23.8)	14.2 (11.6 to 17.3)	15.5 (12.9 to 18.5)	35.3 (21.2 to 58.7)	50.0 (31.3 to 79.9)
Substance use disorder	38.1 (26.3 to 55.3)	31.0 (24.1 to 39.7)	71.8 (55.6 to 92.7)	36.0 (31.7 to 40.8)	49.8 (40.3 to 61.6)	22.5 (20.2 to 25.1)	21.0 (15.9 to 27.6)	12.6 (10.7 to 14.8)
Psychotropic medication								
Antidepressant	15.0 (12.0 to 18.6)	12.0 (10.3 to 14.1)	14.0 (12.4 to 15.7)	12.9 (11.9 to 13.9)	11.3 (10.2 to 12.4)	9.1 (8.5 to 9.7)	6.4 (5.7 to 7.1)	5.7 (5.3 to 6.2)
Antipsychotic	29.0 (20.6 to 40.8)	22.4 (17.6 to 28.7)	37.1 (31.1 to 44.2)	28.3 (25.1 to 31.8)	19.4 (16.9 to 22.2)	12.1 (11.0 to 13.4)	9.0 (7.7 to 10.6)	6.1 (5.3 to 6.9)
Psycho stimulants	7.5 (5.4 to 10.3)	4.0 (3.1 to 5.1)	11.2 (9 to 13.9)	9.7 (8.4 to 11.2)	8.2 (6.2 to 10.8)	6.8 (5.5 to 8.4)	2.0 (0.8 to 5.2)	5.6 (3.2 to 9.8)
Sedatives	17.1 (13.6 to 21.4)	11.9 (10.2 to 14.0)	22.7 (19.9 to 25.8)	16 (14.8 to 17.4)	17.2 (15.4 to 19.2)	11.3 (10.5 to 12.1)	8.4 (7.4 to 9.6)	5.7 (5.3 to 6.1)
Somatic conditions								
Aggressive cancers	NA†	NA†	NA†	NA†	3.1 (1.0 to 9.4)	4.5 (2.0 to 10.6)	4.0 (2.2 to 7.1)	5.2 (3.5 to 7.7)
Amyotrophic lateral sclerosis	NA†	NA†	NA†	NA†	NA†	4.3 (1.1 to 16.6)	40.0 (4.5 to 357.9)	6.2 (2.6 to 14.8)
Dementia or delirium	30.0 (3.1 to 288)	12.0 (3.7 to 39.3)	33.3 (13.4 to 83.0)	7.2 (4.2 to 12.3)	12.6 (7.6 to 20.9)	10.5 (7.5 to 14.8)	2.7 (2.1 to 3.5)	2.0 (1.7 to 2.5)
Bereavement								
Death of a first degree relative	2.4 (0.8 to 7.1)	2.7 (1.6 to 4.8)	2.5 (1.8 to 3.3)	1.8 (1.4 to 2.2)	1.2 (1.0 to 1.5)	1.4 (1.2 to 1.5)	1.2 (0.9 to 1.6)	0.9 (0.7 to 1.1)
Death of a child (<18 y)	NA†	NA†	13.3 (3.0 to 59.6)	5.4 (2.1 to 13.5)	20.0 (1.8 to 221)	26.7 (7.1 to 101)	NA†	10.0 (0.6 to 160)
Death of a child (>18 y)	NA†	NA†	NA†	NA†	8.8 (4.3 to 17.9)	3.2 (1.5 to 6.9)	2.4 (1.3 to 4.4)	1.3 (0.7 to 2.2)
Sociodemographic factors‡								
Disability pension	8.9 (6.3 to 12.5)	2.4 (1.8 to 3.1)	10.8 (9.3 to 12.6)	6.6 (5.9 to 7.3)	5.9 (5.4 to 6.5)	4.3 (4.0 to 4.7)	5.3 (4.0 to 7.2)	3.3 (2.7 to 4.1)
Divorce	1.4 (0.2 to 12.2)	5.0 (0.9 to 27.3)	2.5 (1.8 to 3.5)	2.0 (1.6 to 2.7)	3.0 (2.2 to 4.2)	2.9 (2.4 to 3.6)	3.8 (1.4 to 10.8)	3.4 (2.1 to 5.7)
Long-term sickness absence	5.5 (3.0 to 10.2)	7.7 (4.7 to 12.6)	8.6 (7.4 to 10.0)	9.6 (8.5 to 11.0)	4.4 (3.9 to 5.0)	4.4 (3.9 to 4.8)	NA§	NA§
Relative poverty	1.8 (1.5 to 2.3)	1.5 (1.3 to 1.7)	5.2 (4.7 to 5.9)	3.7 (3.4 to 3.9)	6.1 (5.5 to 6.7)	4.5 (4.2 to 4.8)	2.4 (2.2 to 2.7)	2.5 (2.4 to 2.7)
Social benefits	3.8 (3.0 to 4.8)	2.9 (2.5 to 3.4)	6.2 (5.4 to 7.1)	6.0 (5.5 to 6.6)	3.5 (3.0 to 4.0)	3.4 (3.1 to 3.8)	NA§	NA§
Unemployment	3.2 (1.4 to 7.6)	2.1 (1.3 to 3.3)	1.7 (1.3 to 2.1)	2.2 (1.9 to 2.5)	1.7 (1.4 to 2.2)	1.9 (1.7 to 2.2)	NA§	NA§

* Conditional on age (in years), sex and county of residence by matching.

† Risk indicators too rare to estimate associations.

‡ Excludes people aged 15 years since prior year information was not available until age 16 years.

§ Risk indicators related to working life excluded for people aged 65+ years.

Most risk indicators showed stronger associations with suicide among females on the relative risk scale (figure 1**,[Table T3]** table 3). The median (IQR) point OR of the 25 risk indicators was 14.6 (5.3, 29.1) for females and 10.3 (4.3, 21.3) for males. Further, associations generally peaked among people aged 25–44 years and declined in older age, with median (IQR) ORs of 15.4 (4.3, 26.7) for 15–24 year-olds, 17.4 (6.5, 29.0) for 25–44 year-olds, 11.3 (4.4, 20.0) for 45–64 year-olds and 8.0 (3.6, 23.7) for people aged 65+years (figure 1**,[Table T3]** table 3). There are some exceptions to this pattern: (1) self-harm showed stronger associations with suicide among females in general, but with the strongest association found among males 65 years or older; (2) for depressive and anxiety disorders, associations with suicide were rather similar across sex and age groups; (4) stress-related disorders varied in their associations with suicide across sex and age groups but with no clear pattern; (5) bereavement of different kinds showed no apparent sex differences, but associations were somewhat stronger in younger age groups; (6) divorce showed no apparent sex or age pattern in its association with suicide; (7) long-term sickness absence and unemployment showed no clear sex differences in their associations with suicide (table 3). Many sex-specific and age-specific differences in specific risk indicators are uncertain, however, with overlapping CIs.

On the risk difference scale, nearly all risk indicators showed stronger associations with suicide among males across age groups. Self-harm, and all mental disorders and psychotropic medications, and most sociodemographic factors showed larger risk differences among males than females across age groups. The median (IQR) point risk difference for the 25 risk indicators was 104 (33, 226) per 100 000 person-years for females and 176 (73, 419) for males. Risk differences peaked among 25–44 year-olds, with median (IQR) point risk difference of 124 (37, 260) in 15–24 year-olds, 166 (70, 394) in 25–44 year-olds, 163 (65, 341) in 45–64 year-olds and 138 (31, 356) in people aged 65+ years (online supplemental eTable 3**,[Fig F1]** figure 1).

#### Sensitivity analysis

Excluding risk indicators occurring in the week prior to the index date gave largely similar estimates for psychiatric inpatient and outpatient care and all mental disorders. For self-harm, however, the OR was reduced by 18% in the full sample but remained the strongest association (online supplemental eTable 2 and 5).

## Discussion

We conducted a nested case-control study of 19 741 suicide cases and 197 296 general population controls to describe the sex-specific and age-specific prevalence of 25 clinical and sociodemographic risk indicators in the year preceding suicide and their associations with suicide in the general population. Importantly, we aimed to describe risk indicators, not causes, of suicide. This comprehensive account of sex-specific and age-specific short-term suicide risk indicators allowed us to discern some key patterns not easily recognised in prior research.

First, nearly all risk indicators were less prevalent preceding male suicides, compared with female suicides, and most indicators also had weaker associations with suicide among males than females, on the relative risk scale. This sex difference is noteworthy as it suggests that most risk indicators have a lower statistical sensitivity for predicting suicide in males, meaning that a lesser proportion of male suicides could be detected using these register-based indicators. One possible explanation for this, at least for risk indicators related to psychiatric care, is that men facing mental health problems do not seek care to the same extent women do.^21^ This could explain the low prevalence and could also attenuate the associations between mental disorders and care utilisation with suicide among males, similar to how misclassification can lead to underestimated effect sizes. Another explanation could be sex differences in the methods of suicide. Males who die by suicide tend to use more lethal methods than females,^22^ which could partly explain why register-based risk indicators are less frequent among males who die by suicide. Our results dovetail some prior findings showing that prediction models, using data from mainly electronic health records but also other registers, show lower sensitivities for predicting suicide and suicide attempts among males than females,^23 24^ even when sex is accounted for.

Second, most risk indicators were associated with greater risk differences and a higher absolute suicide risk among males. Simply put, although it was less likely that a male who died by suicide would have a given risk indicator, males that did have the indicator were generally at a higher absolute risk of suicide than females with the same indicator. These opposing sex differences in the relative versus absolute suicide risk may seem contradictory. They are explained, however, by the higher rates of suicide among males. For example, we found a three-fold elevation in suicide risk the year following divorce for both females and males aged 45–64 years. Since the suicide rate is twice as high among males, this three-fold elevation translates to 28 additional suicides among females and 56 additional suicides among males per 100 000 person-years. From a public health perspective, such absolute suicide rates are important to identify groups for whom preventive public health efforts may be especially warranted. Such groups include older males presenting with self-harm, who showed the highest suicide rates of all strata under investigation, although suicide rates were highly elevated following self-harm in all groups. Another group with particularly elevated suicide rates was older males with stress-related disorders.

Third, both the prevalence of risk indicators prior to suicide and the magnitude of their association with suicide generally peaked in middle age and attenuated in older age. Hence, fewer suicides among older people could be detected using register-based indicators. There were some exceptions to this pattern. Interestingly, the prevalence of psychotropic medications was similar in older versus middle-aged individuals who died by suicide, but their associations with suicide were weaker in older people, especially for antidepressants and sedatives. Our results are descriptive and do not imply causality, but we speculate that these findings may be explained by differences in prescribing patterns across age groups. Chronic pain, rather than depression, has been identified as the most common indication for antidepressant medication among individuals over 65 years of age,^25^ which could explain why psychotropic medications are more prevalent and less indicative of suicide in older people. Further, as expected, the prevalence of both dementia/delirium and bereavement increased with age, but their associations with suicide were stronger in the younger age groups compared with older groups, mirroring prior findings that suicide risk is particularly high after a dementia diagnosis^26^ and bereavement^27^ below age 65 years. This could potentially be explained by dementia and bereavement being less expected and more tragic at younger ages, thus affecting the risk of suicide to a greater degree. However, given the descriptive nature of our analyses, we are not able to infer causality.

Our study makes several contributions. First, it provides a comprehensive account showing considerable variability in short-term suicide risk indicators across sex and age groups. This variability may be hard for clinicians to integrate into clinical risk formulations, highlighting the need for structured approaches to suicide risk assessments, such as clinical prediction models taking sex-specific and age-specific risk indicators into account.^28^ Second, by covering a range of different risk indicators, our study allowed us to discern some general patterns in suicide risk indicator across sex and age groups, not easily recognised from prior research. Our findings indicate that suicides among males and older people may be harder to predict, since many occur without being preceded by strong risk indicators, such as self-harm or diagnosed mental disorders. Prevention efforts that go beyond psychiatric settings may therefore be of particular importance to detect males and older people at high risk of suicide. This could include suicide screening followed by safety planning in primary care, which has been shown to reduce suicide attempts.^29^ Males who do present with risk indicators, however, pose important targets for suicide prevention efforts, given their high absolute suicide rates. Such efforts could include, for instance, safety planning with telephone follow-up for suicidal patients, which has shown promising results preventing suicidal behaviour following emergency visits in predominantly male samples.^30^

### Strengths and limitations

This nationally representative study covered all suicides in individuals aged 15 years or older in Sweden from 2009 to 2021. The large sample size enabled stratified analyses with reasonable precision even when combining a rare outcome with rare exposures. The information contained in the Swedish registers allowed for studying a comprehensive set of indicators without recall or self-reporting bias. The case-control design is an efficient design for estimating risk indicators for suicide over the relatively short period of 1 year. This short-term perspective on suicide risk indicators is largely missing in prior literature.^14^

Our study also has several limitations. First, although our 1-year exposure window provides a shorter time window than most prior studies, even shorter time windows of weeks, days or hours would be desirable to guide clinical action to prevent suicide.^14^ We chose a 1-year exposure window to capture short-term risk while retaining enough statistical power to estimate associations. Second, the NPR only contains data on healthcare visits and diagnoses registered by physicians in inpatient and specialised outpatient care, excluding primary care and other professions. Our estimates for different mental disorder categories are therefore not likely to generalise to primary care contexts. However, risk estimates related to psychotropic medications, which use data from the PDR that captures all dispensed medications across the country regardless of the prescriber, can be viewed as a proxy for mental disorders in both primary and specialised care. Our findings for the psychotropic medications show the same general risk indicator pattern as for most mental disorders, with weaker relative risks for risk indicators among males and older persons. Third, our selection of risk indicators was based on not only prior research but also register availability and naturally does not cover all possible risk indicators. Fourth, although our sensitivity analysis suggests that the association between self-harm and suicide may be somewhat inflated, it remained the strongest risk indicator for suicide. Fifth, case-control designs are sometimes considered suboptimal to cohort designs. However, nested case-control studies with incidence density sampling provide estimates largely equivalent to those from cohort studies for time-varying exposures.^19^ Still, since controls were matched to cases on sex, age and county of residence, we could not provide estimates of suicide risk for the sex and age variables. Sixth, importantly, our results are descriptive. Many of the indicators studied here, including psychiatric treatment and medications, should be understood as markers of distress and not as causes of suicide. In line with the descriptive aims of the paper, estimates were not mutually adjusted for other risk indicators and estimates are therefore not independent of each other. This is especially important for the interpretation of risk indicators related to psychiatric care, mental disorders and psychotropic medications, which commonly co-occurred within the same individuals (online supplemental eFigure 1).

## Conclusions

We found considerable heterogeneity in the prevalence of risk indicators preceding suicide, and in their associations with suicide, across sex and age groups. Risk indicators were generally less common and displayed weaker associations with suicide on the relative risk scale among males and older people, suggesting that suicides in these groups will be harder to predict as indicators are rarer. When males did present with risk indicators, however, they generally had a higher absolute risk of suicide, making them important targets for preventive efforts even when risk indicators do not cause suicide. Our findings underscore the importance of considering sex-specific and age-specific risk indicators for individualised suicide prediction and prevention.

## Supplementary material

10.1136/bmjment-2025-301959 online supplemental file 1

## Data Availability

Data may be obtained from a third party and are not publicly available.
