# Supplementary material for: Patterns of sex-specific and age-specific risk indicators of suicide: a population-nested case-control study
Source: BMJ Ment Health. 2025 Oct 28;28(1):e301959. doi: 10.1136/bmjment-2025-301959 (PMC12570955; doi:10.1136/bmjment-2025-301959)
Supplement: online supplemental file 1 [file bmjment-28-1-s001.pdf]

**SUPPLEMENTARY MATERIAL**

Johansson, F., Gunnarsson, L., Grossmann, L., Mataix-Cols, D., Fernández de la Cruz, L., Fazel, S., Gardner, R. M., Dalman, C., Wallert, J. & Rück, C. Patterns of sex- and age-specific risk indicators of suicide: a population nested case-control study.

**eTable 1.** Risk indicator definitions (p. 2).

**Changes from preregistration** (p.3)

**eTable 2.** Occurrence of risk indicators in the prior year and odds ratios of death by suicide in the general population (p.4)

**eTable 3.** Suicide rate differences per 100 000 person-years of between exposed and unexposed conditional on sex and age groups in the general population (p. 5)

**eTable 4.** Prevalence of specific risk indicators in the prior year among 197 296 general population controls (p. 6-7)

**eTable 5.** Sensitivity analysis excluding risk indicator occurring in the week prior to the index date (p. 8)

**eFigure 1.** Correlation matrix of phi-correlations between risk indicators (p. 9)

**References** (p. 10)

| **eTable 1.** Risk indicator definitions | | |
|---|---|---|
| **Risk indicator** | **Definition** | **Data source** |
| **Self-harm and psychiatric contacts** | | |
| Self-harm | ICD-10: X60-X84 | National Patient Register |
| Psychiatric inpatient care | Admission to inpatient psychiatric care | National Patient Register |
| Psychiatric outpatient care | Any contact with outpatient psychiatric care as registered in the NPR | National Patient Register |
| **Mental disorders** | | |
| Anxiety disorder | ICD-10: F40-41 | National Patient Register |
| Depressive disorder | ICD-10: F32-39 excl. F32.3 | National Patient Register |
| Personality disorder | ICD-10: F60-61 | National Patient Register |
| Psychotic disorder | ICD-10: F20-F29 | National Patient Register |
| Stress-related disorder | ICD-10: F43 | National Patient Register |
| Substance-use disorder | ICD-10: F10-19 | National Patient Register |
| **Psychotropic medication** | | |
| Anti-depressant | ATC code: N06A | Prescribed Drug Register |
| Anti-psychotics | ATC code: N05A | Prescribed Drug Register |
| Psychostimulants | ATC code: N06B | Prescribed Drug Register |
| Sedatives | ATC code: N05B and N05C | Prescribed Drug Register |
| **Somatic conditions** | | |
| Aggressive cancers | ICD-10: C15, C22-C25, C33-C34, C56, C76, C80 (see supplement in [1] for definition of aggressive cancers) | Cancer registers |
| ALS | ICD-10: G12.2 [2] | National Patient Register |
| Dementia or delirium (not induced by alcohol or drugs) | ICD-10: F00-F09, G30-31 definition taken from [3]. | National Patient Register |
| **Bereavement** | | |
| Death of a 1st degree relative | Death of parent, sibling (including half siblings and adopted siblings) and/or child (above age 18) | Cause of Death Register, Multi-generation register |
| Death of a child (< 18 y) | | Cause of Death Register, Multi-generation register |
| Death of a child (> 18 y) | | Cause of Death Register, Multi-generation register |
| **Sociodemographic factors** | | |
| Divorce | A change in civil status from married or registered as partner to separated in the prior calendar year | LISA |
| Disability pension | Receipt of disability pension | LISA |
| Long-term sickness absence | Sickness absence for more than 90 net days | LISA |
| Relative poverty | Disposable family income of 60% or less than the annual median income in Sweden | LISA |
| Social benefits | Living in a household receiving social benefits | LISA |
| Unemployment | Registered as full-time unemployed for more than 180 days. Individuals enrolled in | LISA |

| | | |
|---|---|---|
| | education are not classified as unemployed. | |
| ALS: Amyotrophic lateral sclerosis<br>LISA: the Longitudinal Integrated Database for Health Insurance and Labour Market Studies | | |

**Changes from pre-registration**

The pre-registered analytic plan was designed before conducting any analyses and is available at https://osf.io/2dv3f.

Changes from the preregistration were:

1. The study period was changed from 2009-2023 to 2009-2021 since we did not have access to data from the total population register after 2021.
2. Estimates of risk differences were not specified in the pre-registration but were added during analysis.
3. We decided not to use composite variables of all risk indicators in a given domain, as they were considered too broad and heterogenous.
4. The variable social benefits originally included housing benefits. Housing benefits were removed as data were missing on housing benefits for the last three years (2018-2021).

| **eTable 2.** Occurrence of risk indicators in the prior year and odds ratios of death by suicide in the general population | | | | |
|---|---|---|---|---|
| | **Cases, n=19 741, n (%)** | **Controls n=197 296, n (%)** | **OR [a] (95% CI)** | **OR [b] (95% CI)** |
| **Self-harm and psychiatric contact** | | | | |
| Self-harm | 2007 (10.2) | 229 (0.1) | 100.1 (86.5-115.8) | 82.8 (71.5-95.8) |
| Psychiatric inpatient care | 4891 (24.8) | 1234 (0.6) | 53.7 (49.9-57.7) | 50 (46.6-53.8) |
| Psychiatric outpatient care | 8220 (41.6) | 6619 (3.4) | 22.8 (21.9-23.8) | 21.7 (20.8-22.7) |
| **Mental disorders** | | | | |
| Anxiety disorder | 3257 (16.5) | 1948 (1) | 21.3 (20-22.7) | 20.4 (19.2-21.7) |
| Depressive disorder | 3615 (18.3) | 2089 (1.1) | 21.6 (20.3-22.9) | 20.5 (19.3-21.7) |
| Personality disorder | 1036 (5.2) | 362 (0.2) | 31.6 (27.9-35.8) | 30.8 (27.1-34.9) |
| Psychotic disorder | 1245 (6.3) | 900 (0.5) | 14.9 (13.6-16.2) | 14.6 (13.4-16.0) |
| Stress-related disorder | 1465 (7.4) | 859 (0.4) | 19 (17.4-20.8) | 17.8 (16.3-19.6) |
| Substance use disorder | 4190 (21.2) | 1886 (1) | 28.9 (27.2-30.8) | 28.7 (26.1-29.5) |
| **Psychotropic medication** | | | | |
| Anti-depressant | 9621 (48.7) | 19497 (9.9) | 9.4 (9.1-9.7) | - |
| Anti-psychotic | 4453 (22.6) | 3586 (1.8) | 15.7 (15-16.5) | - |
| Psycho stimulants | 999 (5.1) | 1384 (0.7) | 7.8 (7.2-8.5) | - |
| Sedatives | 11333 (57.4) | 24326 (12.3) | 11.6 (11.2-12) | - |
| **Somatic conditions** | | | | |
| Aggressive cancers | 69 (0.3) | 148 (0.1) | 4.7 (3.5-6.3) | - |
| ALS | 15 (0.1) | 23 (0) | 6.5 (3.4-12.5) | - |
| Dementia or delirium | 361 (1.8) | 1100 (0.6) | 3.5 (3.1-4) | - |
| **Bereavement** | | | | |
| Death 1st degree relative | 777 (3.9) | 5932 (3) | 1.3 (1.2-1.4) | - |
| Death of a child (<18y) | 22 (0.1) | 23 (0) | 9.6 (5.3-17.2) | - |
| Death of a child (>18y) | 51 (0.3) | 199 (0.1) | 2.6 (1.9-3.5) | - |
| **Sociodemographic factors [c]** | | | | |
| Disability pension | 3773 (19.1) | 10305 (5.2) | 5.3 (5.1-5.6) | - |
| Divorce | 296 (1.5) | 1127 (0.6) | 2.7 (2.3-3) | - |
| Long-term sickness absence | 1910 (9.7) | 3927 (2) | 5.6 (5.3-6) | - |
| Relative poverty | 9988 (50.6) | 47974 (24.3) | 3.6 (3.5-3.7) | - |
| Social benefits | 2597 (13.2) | 7523 (3.8) | 4 (3.8-4.2) | - |
| Unemployment | 681 (3.4) | 3611 (1.8) | 1.9 (1.8-2.1) | - |

[a] Conditional on age (in years), sex and county of residence by matching
[b] Sensitivity analysis excluding risk indicators occurring within one week of the index date (suicide or sampling)
[c] Excludes persons of age 15 since prior year information was not available.

**eTable 3.** Suicide rate differences per 100 000 person-years of between exposed and unexposed conditional on sex and age groups in the general population

| | Risk difference [a] (95% CI) | | | | | | | |
|---|---|---|---|---|---|---|---|---|
| | **15-24 y** | | **25-44 y** | | **45-64 y** | | **65+ y** | |
| | **Female** | **Male** | **Female** | **Male** | **Female** | **Male** | **Female** | **Male** |
| **Psychiatric contacts and self-harm** | | | | | | | | |
| Self-harm | 424.6 | 729.9 | 1266.7 | 1805.3 | 1398.2 | 2184.4 | 1719.6 | 6991 |
| Psychiatric inpatient care | 354.1 | 624.4 | 530.7 | 985.2 | 729.8 | 863.3 | 469.3 | 1140 |
| Psychiatric outpatient care | 68.9 | 139.6 | 127.7 | 310.8 | 201 | 341.1 | 178.9 | 414.4 |
| **Mental disorders** | | | | | | | | |
| Anxiety disorders | 93.6 | 296.5 | 178.7 | 388.1 | 215.5 | 506.4 | 218.4 | 556 |
| Depressive disorders | 109.2 | 218.4 | 132.3 | 421.3 | 248 | 478.7 | 241.2 | 661.3 |
| Personality disorders | 270.9 | 420.8 | 359.1 | 617.5 | 376.4 | 584 | 341.3 | 389.9 |
| Psychotic disorders | 193.1 | 539.6 | 268.3 | 485.1 | 164.6 | 233.9 | 114.7 | 207.4 |
| Stress-related disorders | 159.2 | 293.5 | 168.3 | 412.3 | 169.8 | 410.5 | 369.8 | 1432.5 |
| Substance use disorders | 214.5 | 409.9 | 493.8 | 601.8 | 538.6 | 507.1 | 203.2 | 319.5 |
| **Psychotropic medication** | | | | | | | | |
| Anti-depressant | 54.4 | 133.8 | 50.2 | 163.7 | 57.4 | 149 | 30.6 | 95.2 |
| Anti-psychotic | 163.2 | 309.2 | 226.6 | 474.7 | 187.9 | 275.4 | 73.6 | 139.1 |
| Psycho stimulants | 44.9 | 49.2 | 91.8 | 189.2 | 98.9 | 170.3 | 11.2 | 137.9 |
| Sedatives | 64.1 | 133.3 | 72.5 | 192 | 64.5 | 160.7 | 26.3 | 77.1 |
| **Somatic conditions** | | | | | | | | |
| Aggressive cancers | NA | NA | NA | NA | 29.9 | 105.3 | 33.5 | 125.3 |
| ALS | NA | NA | NA | NA | NA | 99.3 | 437.5 | 156.2 |
| Dementia or delirium | 225.4 | 190.5 | 319.6 | 146.9 | 163.2 | 282.5 | 18.5 | 29.6 |
| **Bereavement** | | | | | | | | |
| Death 1st degree relative | 10.9 | 29.3 | 14.7 | 18.9 | 2.8 | 11.8 | 2.2 | -3 |
| Death of a child (<18y) | NA | NA | 122.9 | 104.6 | 270.7 | 772.7 | NA | 270.8 |
| Death of a child (>18y) | NA | NA | NA | NA | 110.6 | 66.2 | 15.7 | 9 |
| **Sociodemographic factors [b]** | | | | | | | | |
| Disability pension | 54.2 | 23.6 | 77 | 117.1 | 43.7 | 78.6 | 44.9 | 66.8 |
| Divorce | 3.1 | 69.5 | 14.8 | 23.6 | 28.1 | 56.3 | 31.4 | 72 |
| Long-term sickness absence | 34.2 | 114.2 | 61.2 | 182.5 | 41.7 | 93.4 | NA | NA |
| Relative poverty | 5 | 7.7 | 22.3 | 40.1 | 41 | 67.6 | 9.7 | 31 |
| Social benefits | 18.2 | 28.6 | 41.7 | 96.5 | 32.5 | 66.5 | NA | NA |
| Unemployment | 17 | 18.9 | 6.9 | 27.6 | 9.9 | 26.5 | NA | NA |

[a] Conditional on age (in years). sex and county of residence by matching
[b] Excludes people of age 15 since prior year information was not available.

Risk differences were approximated by transforming odds ratios using formulas presented by Greenland [4]. General population suicide rates for each sex and age strata collected from publicly available CoDR data. We averaged the yearly suicide rate per 100 000 person-years in the full Swedish population 2009-2021 for each age-sex strata (females 15-24 years: 7.8; males 15-24 years: 17.4; females 25-44 years 10.0; males 25-44 years 23.8, females 45-64 years: 14.3; males 45-64 years 30.1, females 65+ years 11.2, males 65+ years 30.1)

| **eTable 4.** Prevalence of specific risk indicators in the prior year among 197 296 general population controls | | | | | | | | |
|---|---|---|---|---|---|---|---|---|
| | **Risk indicator prevalence n, (%)** | | | | | | | |
| | **15-24 y** | | **25-44 y** | | **45-64 y** | | **65+ y** | |
| | **Female (n=5155)** | **Male (n=12136)** | **Female (n=16269)** | **Male (n=40317)** | **Female (n=22790)** | **Male (n=48860)** | **Female (n=15970)** | **Male (n=35799)** |
| **Self-harm and psychiatric contact** | | | | | | | | |
| Self-harm | 24 (0.5) | 26 (0.2) | 30 (0.2) | 58 (0.1) | 27 (0.1) | 46 (0.1) | 9 (0.1) | 9 (0.0) |
| Psychiatric inpatient care | 46 (0.9) | 80 (0.7) | 127 (0.8) | 292 (0.7) | 118 (0.5) | 376 (0.8) | 75 (0.5) | 120 (0.3) |
| Psychiatric outpatient care | 353 (6.8) | 595 (4.9) | 819 (5.0) | 1586 (3.9) | 843 (3.7) | 1581 (3.2) | 320 (2.0) | 522 (1.5) |
| **Mental disorders** | | | | | | | | |
| Anxiety disorder | 146 (2.8) | 124 (1.0) | 303 (1.9) | 454 (1.1) | 310 (1.4) | 360 (0.7) | 117 (0.7) | 134 (0.4) |
| Depressive disorder | 107 (2.1) | 140 (1.2) | 318 (2.0) | 401 (1.0) | 308 (1.4) | 465 (1.0) | 149 (0.9) | 201 (0.6) |
| Personality disorder | 24 (0.5) | 12 (0.1) | 94 (0.6) | 90 (0.2) | 61 (0.3) | 65 (0.1) | 6 (0.0) | 10 (0.0) |
| Psychotic disorder | 10 (0.2) | 21 (0.2) | 50 (0.3) | 192 (0.5) | 139 (0.6) | 332 (0.7) | 73 (0.5) | 83 (0.2) |
| Stress-related disorder | 40 (0.8) | 38 (0.3) | 159 (1.0) | 171 (0.4) | 190 (0.8) | 219 (0.4) | 21 (0.1) | 21 (0.1) |
| Substance use disorder | 47 (0.9) | 124 (1.0) | 107 (0.7) | 441 (1.1) | 140 (0.6) | 647 (1.3) | 86 (0.5) | 294 (0.8) |
| **Psychotropic medication** | | | | | | | | |
| Anti-depressant | 397 (7.7) | 471 (3.9) | 2053 (12.6) | 2501 (6.2) | 3552 (15.6) | 3885 (8.0) | 2934 (18.4) | 3704 (10.3) |
| Anti-psychotic | 65 (1.3) | 111 (0.9) | 256 (1.6) | 541 (1.3) | 497 (2.2) | 939 (1.9) | 443 (2.8) | 734 (2.1) |
| Psycho stimulants | 106 (2.1) | 252 (2.1) | 177 (1.1) | 448 (1.1) | 123 (0.5) | 219 (0.4) | 25 (0.2) | 34 (0.1) |
| Sedatives | 326 (6.3) | 469 (3.9) | 1514 (9.3) | 2299 (5.7) | 3731 (16.4) | 4482 (9.2) | 4826 (30.2) | 6679 (18.7) |
| **Somatic conditions** | | | | | | | | |
| Aggressive cancers | <10 | <10 | <10 | <10 | 13 (0.1) | 18 (0.0) | 40 (0.3) | 76 (0.2) |
| ALS | <10 | <10 | <10 | <10 | <10 | <10 | <10 | 13 (0.0) |
| Dementia or delirium | <10 | <10 | <10 | 32 (0.1) | 27 (0.1) | 65 (0.1) | 307 (1.9) | 657 (1.8) |
| **Bereavement** | 26 (0.5) | 63 (0.5) | 383 (2.4) | 875 (2.2) | 1237 (5.4) | 2771 (5.7) | 524 (3.3) | 1170 (3.3) |
| Death 1st degree relative | 17 (0.3) | 59 (0.5) | 215 (1.3) | 547 (1.4) | 1087 (4.8) | 2383 (4.9) | 511 (3.2) | 1113 (3.1) |
| Death of a child (<18y) | <10 | <10 | <10 | 13 (0.0) | <10 | <10 | <10 | <10 |

| | | | | | | | | |
|---|---|---|---|---|---|---|---|---|
| Death of a child (>18y) | <10 | <10 | <10 | <10 | 16 (0.1) | 28 (0.1) | 51 (0.3) | 104 (0.3) |
| **Sociodemographic factors** [a] | | | | | | | | |
| Disability pension | 100 (1.9) | 277 (2.3) | 477 (2.9) | 1022 (2.5) | 3040 (13.3) | 4068 (8.3) | 550 (3.4) | 771 (2.2) |
| Divorce | <10 | <10 | 169 (1.0) | 317 (0.8) | 153 (0.7) | 406 (0.8) | 13 (0.1) | 58 (0.2) |
| Long-term sickness absence | 33 (0.6) | 37 (0.3) | 558 (3.4) | 583 (1.4) | 1090 (4.8) | 1402 (2.9) | NA[b] | NA[b] |
| Relative poverty | 1717 (33.3) | 3069 (25.3) | 3836 (23.6) | 9427 (23.4) | 3544 (15.6) | 7890 (16.1) | 7408 (46.4) | 11083 (31.0) |
| Social benefits | 394 (7.6) | 1014 (8.4) | 803 (4.9) | 1943 (4.8) | 904 (4.0) | 1761 (3.6) | NA[b] | NA[b] |
| Unemployment | 23 (0.4) | 109 (0.9) | 438 (2.7) | 1226 (3.0) | 426 (1.9) | 1292 (2.6) | NA[b] | NA[b] |

[a] The LISA register contains information on people from 15 years of age, decedents of age 15 were excluded since prior year information was not available.
[b] Risk indicators related to working life excluded for people 65+ years

**eTable 5.** Sensitivity analysis excluding risk indicator occurring in the week prior to the index date.

| | Odds Ratio [a] (95% CI) | | | | | | | |
|---|---|---|---|---|---|---|---|---|
| | **15-24 y** | | **25-44 y** | | **45-64 y** | | **65+ y** | |
| | **Female** | **Male** | **Female** | **Male** | **Female** | **Male** | **Female** | **Male** |
| **Psychiatric contacts and self-harm** | | | | | | | | |
| Self-harm | 67.2 (41-110.2) | 37.3 (24.2-57.5) | 138.5 (89.9-213.5) | 72.9 (54.3-98) | 94.2 (62.9-141.2) | 64.3 (46.6-88.6) | 136.7 (69.5-268.9) | 189.2 (88.5-404.7) |
| Psychiatric inpatient care | 70 (46.8-104.7) | 43.8 (33.3-57.6) | 79.2 (62.3-100.7) | 56.7 (48.8-66) | 67.6 (54-84.5) | 36 (31.5-41.1) | 45 (34-59.6) | 40.3 (32.4-50.2) |
| Psychiatric outpatient care | 21.5 (17.1-27.1) | 13.4 (11.6-15.6) | 35.5 (30.7-41.1) | 25.4 (23.2-27.8) | 27.8 (24.6-31.5) | 17.5 (16.1-19) | 22.3 (18.8-26.5) | 17.1 (15.1-19.5) |
| **Mental disorders** | | | | | | | | |
| Anxiety disorder | 20.1 (14.9-27.3) | 14.7 (11.6-18.6) | 18.3 (15.4-21.6) | 21.8 (19.1-24.9) | 22.6 (19.3-26.5) | 18.8 (16.6-21.2) | 24.9 (19.9-31.2) | 23.8 (19.9-28.6) |
| Depressive disorder | 42.8 (26.6-68.8) | 25.5 (12.7-51.1) | 44.5 (34.5-57.3) | 27.5 (21.3-35.4) | 28.7 (21.2-38.9) | 20.3 (15-27.5) | 31.7 (12.6-79.3) | 14 (6.2-31.5) |
| Personality disorder | 25 (12-52.1) | 32.3 (19.5-53.3) | 29.1 (20.9-40.5) | 23.9 (19.9-28.7) | 13.3 (10.5-16.8) | 9.1 (7.7-10.7) | 11.4 (8.2-15.7) | 7.6 (5.5-10.7) |
| Psychotic disorder | 23.7 (15.7-35.7) | 17.6 (11.7-26.7) | 20.1 (16.2-25) | 18.8 (15.4-22.9) | 13.7 (11.2-16.7) | 13.7 (11.4-16.4) | 32.4 (19.4-54.1) | 44.3 (27.6-71.1) |
| Stress-related disorder | 37.1 (25.6-53.9) | 28.4 (22.2-36.4) | 67.3 (52.2-86.6) | 34.6 (30.5-39.3) | 46 (37.2-56.9) | 21.7 (19.4-24.2) | 18.9 (14.3-25) | 11.7 (9.9-13.8) |
| Substance use disorder | 67.2 (41-110.2) | 37.3 (24.2-57.5) | 138.5 (89.9-213.5) | 72.9 (54.3-98) | 94.2 (62.9-141.2) | 64.3 (46.6-88.6) | 136.7 (69.5-268.9) | 189.2 (88.5-404.7) |

[a] Conditional on age (in years), sex and county of residence by matching

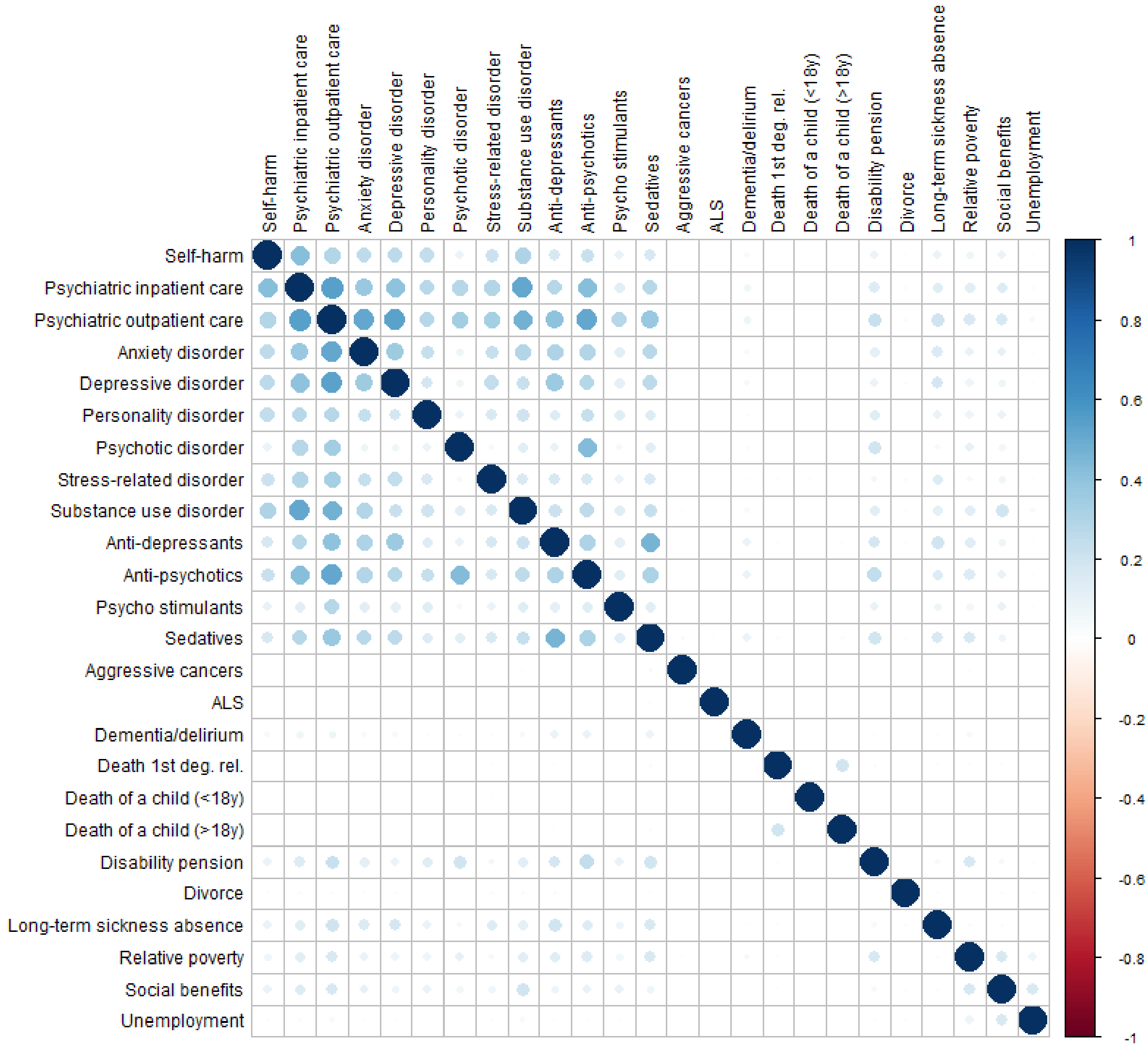


**eFigure 1.** Correlation matrix of phi-correlations between risk indicators in the full sample. The size and color of the dots indicate the strength of the correlations (i.e. co-occurrences) between risk indicators.

**REFERENCES**


1. Liu Q, László KD, Wei D, Yang F, Fall K, Valdimarsdóttir U, et al. Suicide attempt and death by suicide among parents of young individuals with cancer: A population-based study in Denmark and Sweden. PLOS Medicine. 2024 Jan 16;21(1):e1004322.

2. Fang F, Valdimarsdóttir U, Fürst CJ, Hultman C, Fall K, Sparén P, et al. Suicide among patients with amyotrophic lateral sclerosis. Brain. 2008 Oct 1;131(10):2729–33.

3. Garcia-Ptacek S, Kåreholt I, Cermakova P, Rizzuto D, Religa D, Eriksdotter M. Causes of Death According to Death Certificates in Individuals with Dementia: A Cohort from the Swedish Dementia Registry. Journal of the American Geriatrics Society. 2016 Nov 1;64(11):e137–42.

4. Greenland S. Estimation of exposure-specific rates from sparse case-control data. Journal of Chronic Diseases. 1987 Jan 1;40(12):1087–94.
